# Year in review: Liver cancer research in 2022: tumor microenvironment takes the central stage

**DOI:** 10.1097/HC9.0000000000000074

**Published:** 2023-02-27

**Authors:** Weiting Liao, Diego F. Calvisi, Xin Chen

**Affiliations:** 1 Department of Medical Oncology, Cancer Center, West China Hospital, Sichuan University, Chengdu, China; 2 Cancer Biology Program, University of Hawaii Cancer Center, Honolulu, Hawaii; 3 Institute of Pathology, University of Regensburg, Regensburg, Germany

In 2022, significant progress has been made in all aspects of liver cancer research. In particular, immunotherapy-based therapies have become the first-line treatment option for primary liver cancers, including hepatocellular carcinoma (HCC) and intrahepatic cholangiocarcinoma (iCCA). Furthermore, with the advancement of single-cell technologies and multiomics approaches, the comprehensive analysis of the heterogeneous liver tumor microenvironment (TME), especially the tumor immune microenvironment (TIME), has generated significant excitement in the field of liver cancer research.

The immune landscape of liver cancers features a robust suppressive TME. Studies in 2022 focused not only on innate immune cells, such as neutrophils, natural killer cells, and macrophages but also adaptive immune cells, including regulatory T cells (Tregs) and cytotoxic T-lymphocytes in liver TME, shedding light on the complex tumor-immune interaction in mediating tumor progression and therapy resistance. These findings further underpin the clinical imperative for recognizing personalized TME to achieve precise treatment.

Immune profiling of the TIME is essential to elucidate the cellular and molecular mechanisms leading to liver tumor development. Xue et al.^1^ performed single-cell RNA-sequencing analysis of 160 liver tumor samples gathered from 124 treatment-naive patients, including 79 HCCs, 25 iCCAs, and 7 mixed HCC/iCCA samples. Hierarchy clustering identified 5 immune-related cellular modules (CMs). Based on these 5 CMs, patients could be stratified into 5 distinct TIME subtypes: TIME-IA (immune activation), TIME-ISM (immune suppressive myeloid), TIME-ISS (immune suppressive stromal), TIME-IE (immune exclusion), and TIME-IR (immune residence). Detailed analysis revealed that these TIME subtypes exhibited distinct cellular and molecular features and had clinical significance. For instance, high expression of TIME-ISM or TIME-ISS-related CMs was associated with poor progression-free survival. In contrast, high levels of TIME-IR CM predicted better progression-free survival. Intriguingly, tumor-associated neutrophils (TANs) were enriched in the TIME-ISM subtype. Subsequent functional studies indicated that these TANs secrete CCL4 and can recruit macrophages. TANs also showed increased expression of *CD274*, which encodes programmed death-ligand 1, thus suppressing T-cell cytotoxicity. Furthermore, the TANs were conserved in mouse liver tumor models. Notably, neutrophil depletion via Ly6G blockade slowed HCC progression in mice. Altogether, the data revealed that TANs exhibit a gross protumor phenotype, suggesting that the protumor TANs could be promising immunotherapy targets, either alone or in combination with immune checkpoint inhibitors (ICIs).

Another essential cell type in liver TME is the fibroblast or HSC. Indeed, >90% of HCCs develop in a context of fibrosis or cirrhosis. However, the functional contribution of fibroblasts to HCC initiation and progression remains unclear. Filliol et al.^2^ attempted to address this critical issue by combining single-cell RNA sequencing and depleting HSCs in mouse HCC models. The authors found that HSCs promote HCC formation via regulating the premalignant environment and early-stage hepatic carcinogenesis, but have limited roles in modulating late-stage tumors and TIME. Furthermore, the authors discovered that the fibrotic liver contains distinct HSC populations. A dynamic shift between tumor-suppressive and tumor-promoting HSC subpopulations and their mediators may be associated with the risk for HCC formation. Significantly, HGF was enriched in quiescent and cytokine-producing HSCs. These data are consistent with the concept of normal fibroblasts suppressing tumors, referred to as “neighbor suppression.” In contrast, activating HSCs in the fibrotic liver promoted HCC formation *in vivo*. In cirrhosis and fibrosis, characterized by an increased number of HSCs, HGF was presumably upregulated as a protective compensation mechanism, which failed in the late stage. The data suggest that maintaining the balance of HSC or associated mediators in patients with chronic liver disease may reduce the HCC risk.

It is well-established that HCC develops in the background of chronic inflammation, including fatty liver disease, obesity, type II diabetes as well as aggravated exposure to environmental toxins and pollutants. HCC formation is frequently associated with the accumulation of immune cells promoting HCC development via the production of proinflammatory cytokines. In fatty liver disease, excessive circulating free fatty acid in individuals with obesity increased the natural killer cell lipid accumulation and impaired the cytotoxicity against tumor cells. With a potential anti-inflammatory role against protumorigenic inflammation, IL27R signaling promotes HCC, primarily through regulating the NK1.1 innate cytotoxic cells. A recent study revealed that ablation of IL27R or neutralization of IL27R signaling led to decreased HCC formation in DEN-induced and NASH-induced models.^3^ In particular, IL27R signaling within the TIME limited the cytotoxicity of innate cytotoxic lymphocytes. Moreover, IL27R ablation facilitated their accumulation and activation of the innate cytotoxic lymphocytes. Consistently, depletion or functional impairment of innate cytotoxic cells reverted the effect of IL27R disruption. Pharmacologic blockade of IL27 signaling enhanced infiltration of innate cytotoxic lymphocytes and delayed HCC progression. The results suggest that the IL27-dependent strategy regulating innate immune response may have therapeutic potential for patients with chronic liver inflammation who are more susceptible to HCC.

Neutrophils express CXCR2, a receptor critical to neutrophil recruitment in acute injury. It was recently found that CXCR2 is highly expressed in human NASH-related HCCs. In NASH-induced HCC mouse models, mouse lesions were resistant to ICIs, consistent with the clinical observations. Intriguingly, anti–programmed cell death protein 1 (PD1) plus AZD5069, a CXCR2 antagonist, significantly suppressed tumor progression, leading to prolonged survival. Mechanistically, AZD5069 plus anti-PD1 combination therapy increased intratumoral CD8^+^ T-cell numbers and enhanced XCR1^+^ dendritic cell activation. Conversely, genetic suppression of myeloid cell recruitment, neutralization of the XCR1, or depletion of CD8^+^ T cells decreased the therapeutic efficacy of the combination therapy.^4^ Overall, the results suggest that the predominantly immunosuppressive neutrophils can be manipulated to stimulate adaptive tumor immunity, improve responsiveness to ICI-based immunotherapies, and provide novel treatment strategies for NASH-related HCC.

Another study revealed that TIME is regulated by the platelets and how this mechanism contributes to NAFLD/NASH-related HCC development. It was found that platelet depletion accelerated multiple NASH HCC mouse models. Similar results were observed using inhibitors of P2Y12, the major platelet receptor for its activation. Further analysis suggested that platelets released more CD40L, a critical regulator of the adaptive immune response in NAFLD, and P2Y12 inhibitors, such as clopidogrel, could block the increased CD40L in NAFLD. Once released, CD40L bind to its receptor CD40, leading to increased CD4^+^ and CD8^+^ T-cell infiltration in NAFLD liver tissues.^5^ Importantly, the depletion of CD8^+^ T cells, but not CD4^+^ T cells, abolished the P2Y12 inhibitor’s tumor growth-promoting activities *in vivo*. Unlike P2Y12 inhibitors, aspirin, a non-P2Y12 blocking platelet inhibitor, did not accelerate HCC pathogenesis in NAFLD mice. The study uncovered a novel mechanism by which platelet modulates liver TIME and HCC progression, and provides possible guidance on how platelet inhibitors should be administrated in NAFLD patients in clinics.

Mounting evidence indicates that anti-PD1 monotherapy has excellent antitumor efficacy in ∼20% of patients with advanced HCC. To illustrate the molecular mechanism underlying this observation and identify possible biomarkers, Haber et al.^6^ performed an integrated molecular analysis of 111 HCCs treated with anti-PD1. The study identified an 11-gene signature that could predict response and overall survival in HCC patients who received anti-PD1. This gene list included genes involved in interferon-γ signaling, antigen-presenting molecules, and chemotaxis. Interestingly, this 11-gene list has no predictive values in HCC patients pretreated with tyrosine kinase inhibitors (TKIs). The result indicated that TKI treatment might alter the TME, leading to distinct features in HCCs.

Atezolizumab (anti–programmed death-ligand) and bevacizumab (anti-VEGF) combination therapy has become the first-line treatment for advanced HCCs. However, not all patients respond to this therapeutic regimen. Therefore, identifying biomarkers predicting treatment outcomes is critical to select patients who can benefit from this combination therapy. For this purpose, Zhu and colleagues performed an integrated molecular analysis of 358 advanced HCC patients treated with atezolizumab/bevacizumab combination therapy, atezolizumab monotherapy, or sorafenib monotherapy. The study found that preexisting immunity, represented by high expression of CD274, T-effector signature, and intratumoral CD8^+^  T -cell density, was associated with improved survival in patients treated with combination therapy. In contrast, a high Treg to Teff (effective T cells) ratio, and elevated expression of GPC3 and alpha-fetoprotein, were associated with poor clinical outcomes.^7^ The data provide valuable information about possible biomarkers for this first-line therapy against HCC.

Concerning iCCA, Dong et al.^8^ conducted an integrative genomic, transcriptomic, proteomic, phosphoproteomic, and microbiome analysis of 262 Chinese iCCA patients, and identified 4 distinct proteomic subgroups (S1–S4) with subgroup-specific biomarkers. Specifically, S1 was characterized by neutrophil, monocyte, macrophage, dendritic, Th2, and Treg infiltration, mainly related to immunosuppression. S2 displayed instead abundant fibroblasts and endothelial cells, associated with stroma formation and angiogenesis. S4 was enriched in basophils and CD8^+^ naive T cells. Distinct immune checkpoint expression patterns were found among these proteomic subgroups. Thus, designing personalized checkpoint inhibition is necessary for advanced iCCA.

In a separate study, Lin et al.^9^ characterized tumor-immune interactions in human iCCA specimens by analyzing the integrated multiomics data. The study revealed the spatiotemporally heterogeneous immunogenomic features in iCCA. Specifically, 45 human iCCAs were classified into 3 immune groups: sparsely infiltrated with uniformly low immune infiltration across subregions, heterogeneously infiltrated with heterogeneous immune infiltration among subregions, and highly infiltrated with uniformly high immune infiltration across subregions. The study also indicated that iCCA lesions with *KRAS* mutations display more myeloid infiltration but fewer lymphocytes. Also, *BAP1* or *ARID1A* mutations were inversely correlated with neutrophil infiltration, whereas IDH1/2-mutated iCCAs had more T-cell infiltration. FGFR2 alterations (fusion and mutation) were characterized by fewer immune cells, including total TILs, CD4^+^ T cells, and macrophages, lower tumor mutation, or neoantigen burden. Notably, iCCA exhibited an imbalanced CD8^+^ T-cell/Treg population. Tumors with extensive infiltrated immune cells showed immune checkpoint overexpression and CD8^+^ T-cell exhaustion. The lesions also exhibited abundant programmed death-ligand–positive myeloid cells and stromal cells such as cancer-associated fibroblasts. Blocking the cross-talk between immunosuppressive cells and T cell holds promise as a future strategy for iCCA therapies.

Alvisi et al.^10^ reported a study of the comprehensive characterization of T cell and myeloid subgroups in human iCCA through high-dimensional single-cell technologies. Extensive infiltration of CD4^+^ Tregs was observed in conjunction with the loss of CD8^+^ cytotoxic T-lymphocytes. The CD8^+^ T cells expressing CD39, a marker recently related to tumor-specific CD8^+^ T cells, constituted only a minor fraction of iCCA. Furthermore, their abundance was much lower than that in highly immunogenic tumors, such as NSCLC, colorectal cancer, and melanoma. This phenotype may partially explain the relatively low response rate to checkpoint blockade treatment in iCCA patients. Notably, the study showed that tumor Tregs displayed superior immunosuppressive properties. Accordingly, interfering with hyperactivated Tregs to favor T-cell infiltration and function may become a novel direction for iCCA immunotherapy.

In summary, during 2022, significant progress has been made toward the molecular profiling of TIME in HCC and iCCA. The data provide novel mechanistic insight into tumor-environment interactions and how they may contribute to liver tumor development. The results also suggest novel combination therapies against these malignancies. However, their efficacies have yet to be investigated in clinical trials. Furthermore, several studies identified possible biomarkers for immunotherapies. Future studies are required to validate these biomarkers in clinics and illustrate the genes and pathways conferring sensitivity or resistance to immunotherapies.
